# Uniformly positive or negative correlation of cerebral gray matter regions with driving safety behaviors of healthy older drivers

**DOI:** 10.1038/s41598-023-50895-7

**Published:** 2024-01-02

**Authors:** Kaechang Park, Handityo Aulia Putra, Shinichi Yoshida, Fumio Yamashita, Atsushi Kawaguchi

**Affiliations:** 1 Traffic Medicine Laboratory, Research Organization for Regional Alliance, Kochi University of Technology, 185 Miyanokuchi Tosayamada, Kami-Shi, Kochi, 782-0003 Japan; 2 School of Information, Kochi University of Technology, 185 Miyanokuchi Tosayamada, Kami-Shi, Kochi, 782-0003 Japan; 3 Division of Ultrahigh Field MRI, Institute for Biomedical Sciences, Iwate Medical University, Idaidori, Yahaba-Cho, Shiwa-Gun, Iwate, 028-3694 Japan; 4 Education and Research Center for Community Medicine, Faculty of Medicine, Saga University, Saga, 849-8501 Japan

**Keywords:** Neuroscience, Neurology, Engineering

## Abstract

This study investigated the relationship between cerebral gray matter (GM) regions and driving safety behaviors (DSBs) of 98 older drivers without dementia (mean age, 77.72 ± 3.677 years). Their DSBs were evaluated on actual vehicles running on a closed-circuit course. The DSB was scored in six categories: DSB1, visual search behavior; DSB2, speeding; DSB3, signaling of the indicator; DSB4, vehicle stability; DSB5, positioning; and DSB6, steering. The scores were calculated by a single driving instructor; larger scores indicated safer driving performances. Regional GM volumes were measured with voxel-based morphometry by magnetic resonance imaging (MRI). Out of 56 GM regions, 18 were correlated with DSB categories except for DSB4. When a single GM region was correlated with multiple DSB categories, a positive or negative response was uniformly determined for the respective region despite clear differences in the DSB categories. This result suggests the possible existence of two contradictory mechanisms in the brain for DSB. The left postcentral gyrus may largely function in regulating DSBs because it was negatively correlated with five of six DSB categories. Thus, MRI’s measurement of regional GM volumes may help deepen the understanding of the diversity and complexity inherent in brain functions for DSBs.

## Introduction

In most developed countries where the population is aging, road traffic accidents caused by older drivers are increasing annually, and the measurements against the accidents are one of the top priorities, especially in Japan, where the ratio of the population aged ≥ 65 years has reached approximately 30%, and the aging rate is the highest worldwide^1^. The coinciding increase in the number of older drivers with dementia may promote the risk of traffic accidents^2–4^. Nevertheless, the official report released by the Japanese government revealed that more than half of older drivers who had accidents were cognitively normal^5^. In Japan, a cognitive function test has been legally compulsory when renewing a license for older drivers aged ≥ 70 years since 2018. However, excluding older drivers with dementia alone does not completely solve the problem because older drivers without dementia frequently cause traffic accidents^4,6,7^. Traditional measures against traffic accidents include increased awareness of traffic rules, a disciplinary approach to drunk drivers, improved road infrastructure, increased seatbelt use, and enhanced vehicle safety^8,9^. In addition, measures are necessary to deal with driving attributes peculiar to older drivers without dementia, that is, the deterioration of executive function when executing driving safety behaviors (DSBs)^10–12^. It is proposed that DSBs consist of six categories: visual search behavior, speeding, signaling of the indicator, vehicle stability, positioning, and steering^13^.

Since DSBs are controlled by the brain, a research strategy to investigate the brain itself may contribute to developing countermeasures against traffic accidents^13–16^. Although magnetic resonance imaging (MRI) enables one to see inside the brain, its use is limited due to bulky equipment and high cost. Therefore, only two studies have described the relationship between the brain structure data of older drivers and DSBs. First, the Toyota Research Center showed a significantly positive association between the questionnaire scores for DSB and the volume of the supplementary motor area (SMA) in 39 healthy older participants (the SMA volume decreased as DSB scores decreased)^15^. However, they have not evaluated DSB on actual running vehicles and have not reported other cerebral regions. In another study, the Keio University team investigated parts of DSB with machine learning: speeding and vehicle stability at an intersection, in 32 older participants without dementia^16^. They reported that the limited DSBs significantly correlated with the volume of four gray matter (GM) regions, including the left superior part of the precentral sulcus, left sulcus intermedius primus, right orbital part of the inferior frontal gyrus, and right superior frontal sulcus. However, they did not investigate the positive or negative association between DSB score and brain volume as well as other DSB categories, such as positioning and steering, except for an intersection, and various driving locations, such as changing lanes when driving straight and driving a large curve with poor visibility.

Recently, the aging brain, including cerebral atrophy evaluated by MRI, was significantly correlated with the degradation of driving performance when healthy older adults drove motor cars^13^. The evidence indicates a positive correlation between regional GM volume and DSB scores (GM volumes decrease as DSB scores decrease), as brain atrophy results in the shrinking of the whole brain volume. However, no reports on the positive or unexpectedly negative relationship between GM volume and various DSBs in actual vehicle driving exist. In this study, we enrolled 98 older drivers without dementia, examined DSBs representing six categories on actual vehicles running at various driving locations including intersections, and explored how DSBs related to the volume of various brain structures, including the total brain, total GM, total white matter (WM), four cerebral lobes, and 56 GM regions.

## Methods

### Participants

A total of 98 participants (46 men and 52 women; mean age, 77.72 ± 3.677 years) were enrolled in this study. They were recruited from among older people living in the Chugei area of the Kochi prefecture in Japan through local newspapers and television. Each participant received an MRI examination and mini-mental state examinations (MMSE) at Tano Hospital, a medical center in the Chugei area.

To determine the adequacy of the sample size for this study, a power analysis was conducted using random model predictors. The analysis was performed with a predetermined power level of 0.9, an effect size of 0.9, and a significance level of 0.05. The total number of predictors included in the multiple linear regression model was 59, encompassing variables such as age, sex, driving distance, and 56 regional aspects of GM. Using identical parameters for the test predictors, the power analysis, specifically using the Type III F-test, yielded an estimated requisite participant count of 95. Of note, the present study included 98 subjects, surpassing the number determined using the power analysis, thus ensuring a sufficient number for the study.

The average MMSE scores were 28.32 ± 1.590 (range, 24–30; median, 29). A dementia specialist (K.P.) interviewed all participants with their families, examined the participants, and ruled out dementia on the basis of MRI findings, MMSE scores, and neuropsychological tests, such as the trail making test and clock drawing test, including the Conversational Assessment of Neurocognitive Dysfunction, newly developed for dementia diagnosis based on daily conversations^17^. All of them were right-handed and had no cerebrovascular diseases or brain tumors. They also received an evaluation of DSB on actual vehicles running on roads at the Aki Driving School located in the Chugei area of Kochi. The driving experience and exposure of participants were conditioned for the enrollment as follows: all drove > 2 times per week and 5 km per week to work sites, shops, and hospitals. Professional drivers were excluded from the present study.

### Measurement of regional brain volumes

T1-weighted MR images were obtained using the 1.5-Tesla ECHELON Vega system (Hitachi, Tokyo, Japan) with the three-dimensional gradient echo with an inversion recovery sequence. The following scanning parameters were used: repetition time, 9.2 ms; echo time, 4.0 ms; inversion time, 1000 ms; flip angle, 8°; field of view, 240 mm; matrix size, 0.9375 × 0.9375 mm; slice thickness, 1.2 mm; and the number of excitations, 1. Each image was visually assessed for brain diseases and anomalies, head motion, and artifacts affecting the volumetric measurement. We used the VBM8 toolbox (http://dbm.neuro.uni-jena.de/vbm/), which is specially designed for VBM analysis, and other modules implemented in Statistical Parametric Mapping (SPM) 8 (https://www.fil.ion.ucl.ac.uk/spm/) to estimate regional brain volumes. In the toolbox, Diffeomorphic Anatomical Registration Through Exponentiated Lie Algebr*a* (DARTEL) and a custom-made template using IXI Dataset (https://brain-development.org/ixi-dataset/) were used. The DARTEL algorithm has been shown to sensitively detect atrophy in the brain of patients with Alzheimer’s disease^18^.

In brief, the images were segmented into GM, WM, and cerebrospinal fluid space using the maximum a posteriori (MAP) approach^19,20^. The segmented GM and WM images were then used to estimate the morphological correspondence between the template image and the participant’s brain using the high-dimensional nonlinear warping algorithm^21^. The estimated nonlinear warp was inversely applied to an atlas defined in the template space to parcellate the target brain anatomically. The neuromorphometrics atlas incorporated in SPM12 was used for the parcellation, with a modification for WM lesions, which appeared as incorrect GM segments around the lateral ventricles. The volumes of 56 anatomical regions were calculated as the sum of the correspondent tissue densities in the voxels belonging to each region.

### Evaluation by DSBs

**Figure 1:**
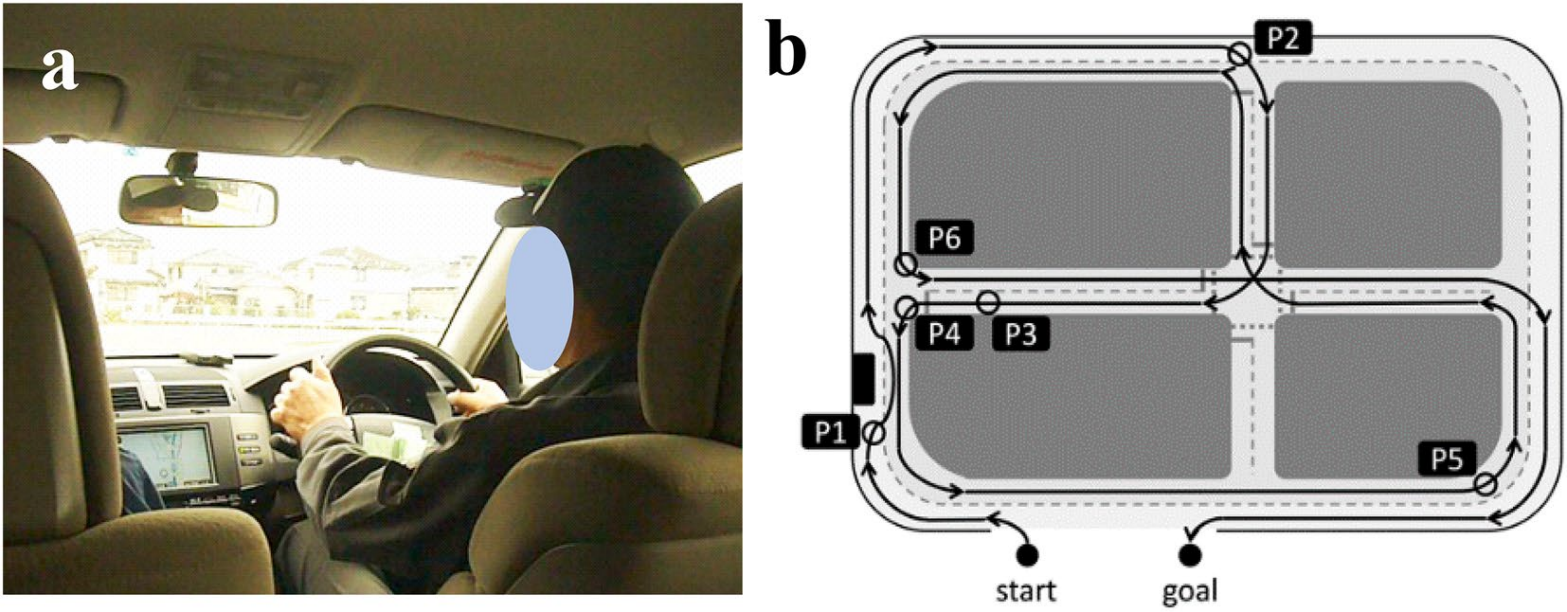
An actual vehicle and a closed-circuit course. (**a**) A view from inside the vehicle. (**b**) Map of the driving course with six rating points. P1, changing line when driving straight; P2, intersection with one right turns; P3, straight course; P4, intersection with one left turn; P5, large curve with poor visibility; P6, another right turn having a stop sign. The corresponding author owns the copyright of the photography.

Actual vehicle driving experiments were performed on a closed-circuit course (Fig. 1a), officially designated for renewing drivers’ licenses for older drivers by the National Police Agency (The Driver’s License Skill Test Implementation Standard), in the Aki Driving School in the Chugei area, Kochi, Japan (Fig. 1a). In the present test, six locations on the driving course were selected for rating. These locations included changing lines when driving straight (Fig. 1b, P1), changing lanes when driving straight; P2, intersection with one right turn; P3, straight course; P4, intersection with one left turn; P5, large curve with poor visibility; P6, another right turn having a stop sign.

A Toyota four-wheel, 1400-cc vehicle (COMFORT) was used. The typical speed of the vehicles on the closed-circuit course ranges from 20 to 50 km/h, and approximately 20 min is taken to complete a circuit. As a good sample of DSB, the same official driving instructor completed the assessment after teaching all participants how to drive. No other driving events were included in the test. In particular, a single instructor drove the course and demonstrated good driving performance once, while participants sat next to him. Then, the participants drove the course once with the instructor sitting on the passenger seat. The instructor assessed each participant’s driving skills at six locations with a 3-point scale based on six category methods described previously^12,13^. They responded to the items using a three-point scale: 1, poorly done; 2, normally done; and 3, well done. These rating scores at six locations were then calculated as the “overall evaluation” by assessing the six categories: “visual search behavior (safety recognition with head movement),” “speeding (choice of vehicle speed),” “signaling (timely and appropriate usage of the indicator),” “vehicle stability (acceleration and braking without knocking and completely pulling up in front of the stop line),” “positioning (vehicle movement along the radius of the curvature at intersections without large or small turns),” and “steering (smooth handling with appropriate starting and ending).” Larger scores indicated stronger compliance with the Road Traffic Act. An average value of the summed scores at the six locations for the two rounds of the course was calculated for the DSBs.

### Statistical analysis

Multivariate linear regression analysis using IBM SPSS Statistics was employed to estimate the impact of various conditions on each DSB category. The dependent variables in the regression models were the DSB categories, whereas the independent variables included driving distance and specific brain regions. Age and gender were included as covariates to account for their potential influence on DSBs.

In this study, 18 regression models were constructed, with 3 regression models corresponding to each DSB category. The DSB category served as the dependent variable in each model. The independent variables for each of the three regression models were as follows: driving distance (weekly driving distance of < 50 km, 1; between 50 and 200 km, 2; between 200 and 500 km, 3; more than 500 km, 4); age; gender (women, 1; men, 0); and brain conditions. Brain conditions consisted of three models: total brain (Table 1), cerebral lobes (Table 2), and 56 specific GM regions (Table 3). The total brain model consisted of total brain, GM, and WM volumes. The cerebral lobes model consisted of the volumes of the frontal, temporal, parietal, and occipital lobes. The GM regions model consisted of the volumes of 56 specific GM regions.

**Table 1:** Coefficients and corresponding significance levels through regression analyses with gray matter, white matter, and total brain volumes.

Explanatory variable	Objective variable—Driving Safety Behavior (DSB)											
	DSB1 ($p$)		DSB2 ($p$)		DSB3 ($p$)		DSB4 ($p$)		DSB5 ($p$)		$DSB6 (p)$	
Sex (Female = 1, Male = 0)	− 0.325 (0.001)	**^bq^	− 0.198 (0.058)		− 0.128 (0.203)		− 0.198 (0.052)		− 0.282 (0.006)	**^bq^	− 0.200 (0.000)	***^bq^
Age	− 0.080 (0.467)		0.043 (0.704)		− 0.166 (0.137)		0.010 (0.930)		0.086 (0.440)		− 0.094 (0.409)	
Driving distance	0.061 (0.537)		0.101 (0.325)		0.132 (0.185)		0.127 (0.205)		0.087 (0.383)		0.061 (0.548)	
Brain region												
Total gray matter	0.073 (0.593)		0.047 (0.738)		− 0.324 (0.019)	*	0.073 (0.598)		0.239 (0.083)		− 0.115 (0.414)	
Total white matter	0.083 (0.520)		− 0.006 (0.963)		− 0.151 (0.249)		0.211 (0.111)		0.169 (0.196)		− 0.020 (0.881)	
Total brain volume	0.070 (0.519)		0.021 (0.852)		− 0.225 (0.046)	*	0.123 (0.274)		0.190 (0.088)		− 0.066 (0.562)	
Statistical model												
*R*^*2*^	0.124		0.050		0.109		0.094		0.108		0.064	
*Adjusted R*^*2*^	0.076		− 0.002		0.061		0.044		0.059		0.013	
*n*	98		98		98		98		98		98	

All volumes were normalized by the intracranial volume for each corresponding participant.

Six DSB scores: DSB1, visual search behavior; DSB2, speeding; DSB3, signaling of the indicator; DSB4, vehicle stability; DSB5, positioning; and DSB6, steering.

*p < 0.05, **p < 0.01, ***p < 0.001.

^b^Significant value after Bonferroni correction.

^q^q < 0.05, the significant value of false discovery rate method.

**Table 2:** Coefficients and corresponding significance levels through regression analyses with frontal, temporal, parietal, and occipital lobes.

Explanatory variable	Objective variable—driving safety behavior (DSB)											
	DSB1 ($p$)		DSB2 ($p$)		DSB3 ($p$)		DSB4 ($p$)		DSB5 ($p$)		DSB6 ($p$)	
Sex (Female = 1, Male = 0)	− 0.416 (0.000)	***^bq^	− 0.202 (0.071)		− 0.119 (0.268)		− 0.234 (0.032)	*	− 0.253 (0.021)	*	− 0.230 (0.040)	*
Age	− 0.083 (0.428)		0.036 (0.750)		− 0.144 (0.187)		− 0.045 (0.683)		0.060 (0.583)		− 0.078 (0.485)	
Driving distance	0.067 (0.500)		0.090 (0.395)		0.109 (0.287)		0.148 (0.151)		0.086 (0.403)		0.069 (0.517)	
Brain region												
Frontal	0.338 (0.032)	*	0.006 (0.971)		− 0.140 (0.385)		0.164 (0.312)		− 0.108 (0.507)		0.082 (0.621)	
Temporal	− 0.206 (0.135)		− 0.061 (0.675)		− 0.169 (0.234)		0.001 (0.996)		0.178 (0.214)		− 0.079 (0.589)	
Parietal	0.032 (0.827)		0.037 (0.814)		− 0.077 (0.613)		− 0.058 (0.701)		0.088 (0.563)		− 0.026 (0.867)	
Occipital	− 0.058 (0.675)		0.075 (0.610)		0.180 (0.208)		− 0.214 (0.135)		0.028 (0.845)		− 0.044 (0.765)	
Statistical model												
*R*^*2*^	0.173		0.057		0.114		0.109		0.105		0.061	
*Adjusted R*^*2*^	0.109		− 0.017		0.045		0.039		0.035		− 0.013	
*n*	98		98		98		98		98		98	

The intracranial volume for each corresponding participant normalized all volumes.

Six DSB scores: DSB1, visual search behavior; DSB2, speeding; DSB3, signaling of the indicator; DSB4, vehicle stability; DSB5, positioning; and DSB6, steering.

*p < 0.05, **p < 0.01, ***p < 0.001.

^b^Significant value after Bonferroni correction.

^q^q < 0.05, the significant value of false discovery rate method.

**Table 3:** Coefficients and corresponding significance levels through regression analyses with 56 regional gray matter volumes.

Explanatory variable	Objective variable—Driving Safety Behavior (DSB)											
	DSB1 ($p$)		DSB2 ($p$)		DSB3 ($p$)		DSB4 ($p$)		DSB5 ($p$)		DSB6 ($p$)	
Sex (Female = 1, Male = 0)	− 0.263 (0.097)		− 0.189 (0.200)		0.166 (0.318)		− 0.107 (0.596)		− 0.252 (0.133)		0.087 (0.638)	
Age	− 0.215 (0.195)		0.091 (0.555)		− 0.122 (0.486)		0.043 (0.840)		0.167 (0.339)		− 0.106 (0.586)	
Driving distance	− 0.039 (0.801)		0.213 (0.145)		− 0.024 (0.882)		0.195 (0.330)		− 0.023 (0.886)		− 0.171 (0.352)	
Brain region												
Right orbital gyrus	0.278 (0.474)		− 0.210 (0.563)		0.312 (0.450)		− 0.133 (0.790)		− 0.591 (0.155)		− 0.081 (0.860)	
Left orbital gyrus	− 0.32 (0.325)		− 0.180 (0.553)		− 0.432 (0.214)		0.077 (0.853)		− 0.008 (0.982)		− 0.066 (0.863)	
Right operculum	0.168 (0.485)		0.033 (0.883)		− 0.100 (0.697)		0.170 (0.585)		− 0.062 (0.806)		0.132 (0.642)	
Left operculum	− 0.257 (0.281)		− 0.042 (0.850)		− 0.061 (0.810)		− 0.215 (0.483)		0.119 (0.637)		0.208 (0.461)	
Right inferior frontal gyrus	0.486 (0.053)		0.582 (0.015)	*	0.085 (0.745)		0.186 (0.556)		0.100 (0.700)		0.132 (0.650)	
Left inferior frontal gyrus	0.078 (0.776)		0.016 (0.949)		− 0.140 (0.631)		− 0.112 (0.751)		0.181 (0.532)		− 0.161 (0.620)	
Right temporal gyrus	− 0.387 (0.370)		0.445 (0.273)		− 0.640 (0.167)		− 0.123 (0.825)		− 0.208 (0.649)		− 1.128 (0.032)	*
Left temporal gyrus	0.812 (0.021)	*	0.369 (0.250)		0.544 (0.137)		0.357 (0.417)		0.551 (0.131)		0.948 (0.023)	*
Right planum polare	− 0.029 (0.910)		0.006 (0.980)		0.322 (0.246)		0.048 (0.885)		0.108 (0.695)		0.016 (0.958)	
Left planum polare	0.108 (0.699)		− 0.287 (0.277)		− 0.064 (0.831)		0.098 (0.785)		− 0.399 (0.182)		− 0.074 (0.823)	
Right occipital gyrus	− 0.275 (0.301)		− 0.280 (0.262)		− 0.054 (0.846)		− 0.029 (0.933)		− 0.106 (0.704)		− 0.222 (0.480)	
Left occipital gyrus	− 0.085 (0.744)		− 0.131 (0.594)		0.604 (0.035)	*	− 0.424 (0.214)		− 0.159 (0.566)		0.089 (0.776)	
Right insula	− 0.698 (0.115)		− 0.643 (0.121)		− 0.600 (0.200)		− 0.077 (0.891)		− 0.854 (0.070)		− 0.589 (0.259)	
Left insula	0.168 (0.688)		0.376 (0.342)		0.545 (0.226)		− 0.154 (0.776)		0.704 (0.119)		− 0.017 (0.972)	
Right cingulate gyrus	− 0.47 (0.071)		− 0.278 (0.250)		0.133 (0.625)		0.030 (0.926)		− 0.098 (0.716)		− 0.381 (0.213)	
Left cingulate gyrus	− 0.068 (0.814)		− 0.396 (0.150)		− 0.029 (0.924)		− 0.181 (0.629)		0.401 (0.197)		0.168 (0.626)	
Right frontal pole	0.117 (0.606)		− 0.185 (0.385)		− 0.147 (0.543)		− 0.064 (0.826)		0.244 (0.312)		− 0.165 (0.539)	
Left frontal pole	0.088 (0.678)		0.103 (0.606)		0.375 (0.103)		− 0.049 (0.859)		− 0.135 (0.548)		0.209 (0.409)	
Right gyrus rectus	− 0.089 (0.746)		− 0.077 (0.767)		− 0.031 (0.915)		0.162 (0.648)		− 0.083 (0.775)		− 0.268 (0.413)	
Left gyrus rectus	0.389 (0.137)		0.230 (0.345)		− 0.076 (0.782)		0.223 (0.505)		− 0.020 (0.943)		0.281 (0.360)	
Right medial frontal cortex	0.449 (0.103)		0.473 (0.068)		0.522 (0.076)		0.036 (0.919)		0.078 (0.787)		0.442 (0.174)	
Left medial frontal cortex	− 0.411 (0.058)		− 0.253 (0.209)		− 0.213 (0.349)		− 0.116 (0.673)		0.253 (0.265)		− 0.313 (0.219)	
Right middle frontal gyrus	0.738 (0.007)	**	0.363 (0.144)		0.465 (0.101)		− 0.211 (0.534)		0.017 (0.950)		0.483 (0.125)	
Left middle frontal gyrus	− 0.902 (0.007)	**	− 0.278 (0.355)		− 0.505 (0.142)		0.185 (0.654)		− 0.275 (0.417)		− 0.468 (0.221)	
Right precentral gyrus	0.375 (0.185)		0.644 (0.018)	*	0.207 (0.488)		0.251 (0.488)		− 0.018 (0.953)		0.496 (0.140)	
Left precentral gyrus	− 0.103 (0.786)		− 1.033 (0.006)	**	− 0.250 (0.538)		− 0.519 (0.294)		− 0.296 (0.464)		− 0.346 (0.444)	
Right subcallosal area	− 0.181 (0.368)		− 0.069 (0.714)		− 0.216 (0.313)		− 0.048 (0.851)		0.026 (0.902)		0.026 (0.913)	
Left subcallosal area	− 0.397 (0.071)		− 0.393 (0.057)		− 0.098 (0.669)		0.027 (0.923)		− 0.195 (0.394)		0.243 (0.344)	
Right superior frontal gyrus	− 0.06 (0.768)		0.309 (0.111)		− 0.312 (0.155)		0.082 (0.755)		0.169 (0.434)		− 0.032 (0.894)	
Left superior frontal gyrus	− 0.096 (0.642)		− 0.070 (0.719)		0.209 (0.346)		0.172 (0.521)		0.272 (0.220)		− 0.170 (0.492)	
Right supplementary motor cortex	0.144 (0.571)		0.437 (0.073)		0.171 (0.529)		0.022 (0.946)		0.245 (0.366)		− 0.260 (0.392)	
Left supplementary motor cortex	0.227 (0.307)		− 0.327 (0.120)		− 0.091 (0.699)		− 0.032 (0.909)		− 0.230 (0.329)		0.322 (0.223)	
Right angular gyrus	− 0.564 (0.029)	*	− 0.584 (0.017)	*	− 0.146 (0.585)		− 0.128 (0.693)		− 0.141 (0.596)		0.230 (0.439)	
Left angular gyrus	0.715 (0.011)	*	0.811 (0.002)	**	− 0.389 (0.177)		0.180 (0.603)		0.348 (0.224)		0.110 (0.729)	
Right precuneus	0.44 (0.197)		0.540 (0.094)		0.445 (0.220)		− 0.371 (0.397)		0.590 (0.105)		0.418 (0.299)	
Left precuneus	− 0.459 (0.169)		− 0.234 (0.450)		− 0.373 (0.292)		0.254 (0.552)		− 0.373 (0.289)		− 0.479 (0.225)	
Right postcentral gyrus	0.303 (0.261)		0.329 (0.195)		0.152 (0.595)		0.327 (0.347)		− 0.018 (0.949)		0.106 (0.739)	
Left postcentral gyrus	− 1.137 (0.000)	***^bq^	− 0.699 (0.010)	**	− 0.667 (0.028)	*	− 0.421 (0.243)		− 0.673 (0.026)	*	− 0.712 (0.035)	*
Right supramarginal gyrus	− 0.373 (0.069)		− 0.557 (0.005)	**	− 0.118 (0.581)		− 0.109 (0.674)		0.054 (0.800)		− 0.258 (0.283)	
Left supramarginal gyrus	0.526 (0.048)	*	0.716 (0.005)	**	0.298 (0.285)		0.217 (0.518)		0.432 (0.122)		0.234 (0.448)	
Right superior parietal lobule	0.217 (0.402)		− 0.153 (0.527)		0.424 (0.127)		0.428 (0.203)		0.318 (0.249)		0.214 (0.484)	
Left superior parietal lobule	− 0.059 (0.797)		− 0.025 (0.908)		− 0.173 (0.477)		− 0.329 (0.268)		− 0.302 (0.216)		− 0.431 (0.116)	
Right entorhinal area	− 0.532 (0.090)		0.056 (0.847)		− 0.187 (0.569)		− 0.336 (0.401)		0.312 (0.343)		0.788 (0.036)	*
Left entorhinal area	0.163 (0.592)		− 0.491 (0.090)		0.223 (0.490)		0.053 (0.891)		− 0.139 (0.666)		− 0.501 (0.168)	
Right fusiform gyrus	− 0.156 (0.586)		− 0.089 (0.740)		0.316 (0.303)		0.276 (0.456)		− 0.118 (0.697)		0.571 (0.098)	
Left fusiform gyrus	0.548 (0.068)		0.570 (0.044)	*	− 0.057 (0.854)		0.089 (0.815)		− 0.110 (0.725)		− 0.487 (0.167)	
Right parahippocampal gyrus	0.607 (0.028)	*	0.653 (0.012)	*	0.363 (0.205)		0.189 (0.583)		0.015 (0.956)		− 0.141 (0.657)	
Left parahippocampal gyrus	− 0.25 (0.442)		− 0.366 (0.232)		− 0.304 (0.380)		0.114 (0.785)		0.080 (0.816)		0.178 (0.643)	
Right calcarine cortex	0.558 (0.127)		0.595 (0.084)		0.059 (0.877)		0.001 (0.998)		0.565 (0.144)		− 0.100 (0.815)	
Left calcarine cortex	− 0.236 (0.489)		0.319 (0.321)		− 0.062 (0.863)		− 0.068 (0.878)		− 0.074 (0.837)		− 0.098 (0.808)	
Right cuneus	− 0.167 (0.539)		− 0.180 (0.480)		− 0.586 (0.047)	*	0.184 (0.599)		− 0.048 (0.867)		0.364 (0.261)	
Left cuneus	0.486 (0.061)		0.056 (0.815)		0.414 (0.131)		0.039 (0.904)		− 0.171 (0.526)		0.326 (0.283)	
Right lingual gyrus	− 0.418 (0.177)		− 0.855 (0.005)	**	0.072 (0.825)		0.058 (0.882)		− 0.469 (0.154)		− 0.140 (0.701)	
Left lingual gyrus	0.092 (0.720)		0.080 (0.738)		− 0.074 (0.784)		− 0.261 (0.430)		0.444 (0.107)		0.109 (0.719)	
Right occipital fusiform gyrus	0.115 (0.566)		0.274 (0.151)		− 0.108 (0.613)		− 0.097 (0.708)		0.187 (0.382)		− 0.014 (0.953)	
Left occipital fusiform gyrus	0.143 (0.449)		0.080 (0.652)		0.032 (0.873)		0.218 (0.373)		0.108 (0.588)		0.146 (0.514)	
Statistical model												
*R*^*2*^	0.691		0.728		0.484		0.652		0.565		0.650	
*Adjusted * *R*^*2*^	0.211		0.306		− 0.318		0.113		− 0.110		0.106	
*n*	98		98		98		98		98		98	

The intracranial volume for each corresponding participant normalized all volumes.

Six DSB scores: DSB1, visual search behavior; DSB2, speeding; DSB3, signaling of the indicator; DSB4, vehicle stability; DSB5, positioning; and DSB6, steering.

*p < 0.05, **p < 0.01, ***p < 0.001.

^b^Significant value after Bonferroni correction.

^q^q < 0.05, the significant value of false discovery rate method.

To determine which cerebral regions are significantly associated with DSBs, we constructed linear regression models for each combination of DSB category and independent variable. Bonferroni’s correction was applied to correct for multiple comparisons in these linear regression analyses.

Statistical significance was defined as a p-value of < 0.05. In addition, variance inflation factors (VIFs) were calculated as a part of the collinearity tests conducted alongside the linear regression models to assess the presence of collinearity among the independent variables.

This comprehensive approach allowed us to examine the relationships among various brain structures, driving distance, and DSBs while controlling for age and gender. The use of multiple regression models, each tailored to specific sets of independent variables, enabled a more nuanced understanding of the factors influencing DSBs across different DSB categories.

The adjusted alpha value for significance was uniformly calculated across all models based on this approach. Specifically:

For each model, irrespective of the number of independent variables it contained (whether 6 as in Table 1, 7 as in Table 2, or 59 as in Table 3), the Bonferroni-corrected significance level was consistently set at 0.05/18 ≈ 0.0028.

This approach ensured a uniform standard for determining statistical significance across all models, thereby mitigating the likelihood of false positives that could arise from multiple testing. Additionally, we have revised our approach to utilize the False Discovery Rate (FDR) method by Benjamini and Hochberg, which better accounts for the interdependencies among multiple variables and provides a more accurate reflection of significant associations in our data. The FDR value is represented as the q-value in Tables 1, 2, and 3.

### Ethics statement

This study was conducted in accordance with the “Ethics Guideline for Medical and Health Research Involving Human Subjects” based on the Declaration of Helsinki. All participants signed a formal agreement outlining that the experimental data would only be used for scientific study and that the results would ensure anonymity. Written informed consent was obtained from all participants. This study was approved by the institutional review board at Kochi University of Technology (Application no. C4-3).

## Results

### Regression with total brain, GM, and WM volumes

We determined the association between DSB scores and independent variables, including total brain volume, GM volume, and WM volume. Six linear regression models were created. DSB1 had an effect size of 0.1417 (p < 0.05), DSB2 had an effect size of 0.0526 (p > 0.05), DSB3 had an effect size of 0.1226 (p > 0.05), DSB4 had an effect size of 0.1032 (p > 0.05), DSB5 had an effect size of 0.1208 (p < 0.05), and DSB6 had an effect size of 0.0681 (p < 0.01). The effect sizes were calculated utilizing Cohen’s f^2^ formula: f^2^ = R^2^/(1 − R^2^), where R^2^ is the coefficient of determination as presented in Tables 1, 2, and 3 for each group of independent variables. This formula was consistently applied across all groups of independent variables to determine their respective effect sizes.

The gender parameter showed a significantly negative correlation with the DSB1, DSB5, and DSB6 scores. In the brain regions, total GM was significantly correlated with DSB2. Males exhibited significantly higher DSB scores in these specific DSB categories. All VIF values for the selected independent variables in the linear regressions remained below 2, indicating an absence of multicollinearity among age, gender, GM, and WM.

### Regression with four cerebral lobes

We explored the relationship between DSB scores and the volume of the four cerebral lobes. Six linear regression models were created. DSB1 had an effect size of 0.2094 (p < 0.05), DSB2 had an effect size of 0.06 (p > 0.05), DSB3 had an effect size of 0.1287 (p > 0.05), DSB4 had an effect size of 0.1219 (p > 0.05), DSB5 had an effect size of 0.1174 (p > 0.05), and DSB6 had an effect size of 0.0645 (p > 0.05). The results are summarized in Table 2.

The gender parameter demonstrated a significant negative correlation with DSB1, DSB4, DSB5, and DSB6, indicating that males exhibited higher DSB scores in these categories. No statistically significant correlations were observed in the four cerebral lobes.

Importantly, all VIF parameters for the selected independent variables used in the linear regressions were below 10, confirming the absence of severe collinearity among age, sex, frontal, temporal, parietal, and occipital lobes.

### Regression with 56 GM regions

Our analysis extended to 56 subdivided GM regions, and the outcomes are summarized in Tables 3 and 4. Six linear regression models were created for the 56 GM regions. DSB1 had an effect size of 2.2349 (p > 0.05), DSB2 had an effect size of 2.6763 (p < 0.05), DSB3 had an effect size of 0.9374 (p > 0.05), DSB4 had an effect size of 1.8775 (p > 0.05), DSB5 had an effect size of 1.299 (p > 0.05), and DSB6 had an effect size of 1.8558 (p > 0.05).

**Table 4:**
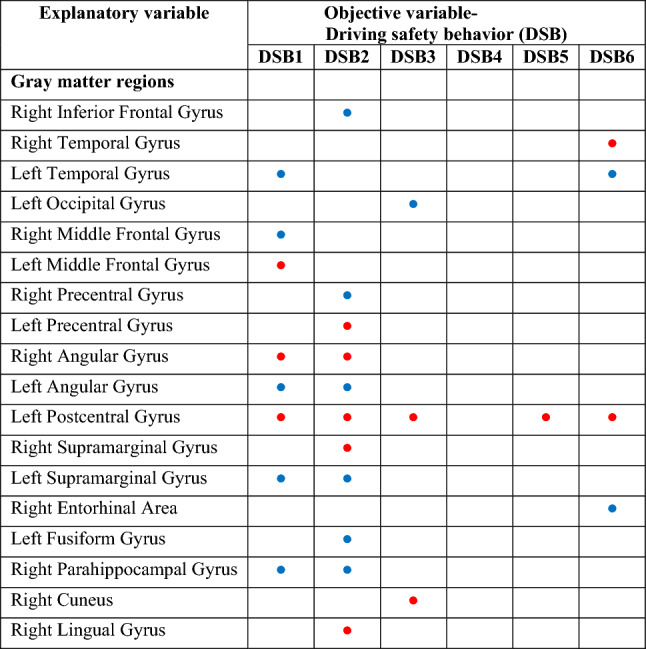
Positive and negative correlations of 18 Gy matter regions with statistical significance (P < 0.05) to driving safety behaviors.

Blue dots, positive estimates; Red dots, negative estimates.

The intracranial volume for each corresponding participant normalized all volumes.

Six DSB scores: DSB1, visual search behavior; DSB2, speeding; DSB3, signaling of the indicator; DSB4, vehicle stability; DSB5, positioning; and DSB6, steering.

Of the 56 GM regions, based on the VIF value, 18 GM regions exhibited statistically significant correlations with some DSB categories, with the exception of DSB4 (Table 3). In some of the 18 GM regions, the VIF exceeded 10. As a result, we conducted a collinearity assessment using these 18 significantly correlated GM regions, revealing VIF parameters all below 8. This supports the assertion that severe collinearity does not exist among age, sex, and the 18 GM regions, as indicated in Supplemental Table 1.

**Figure 2:**
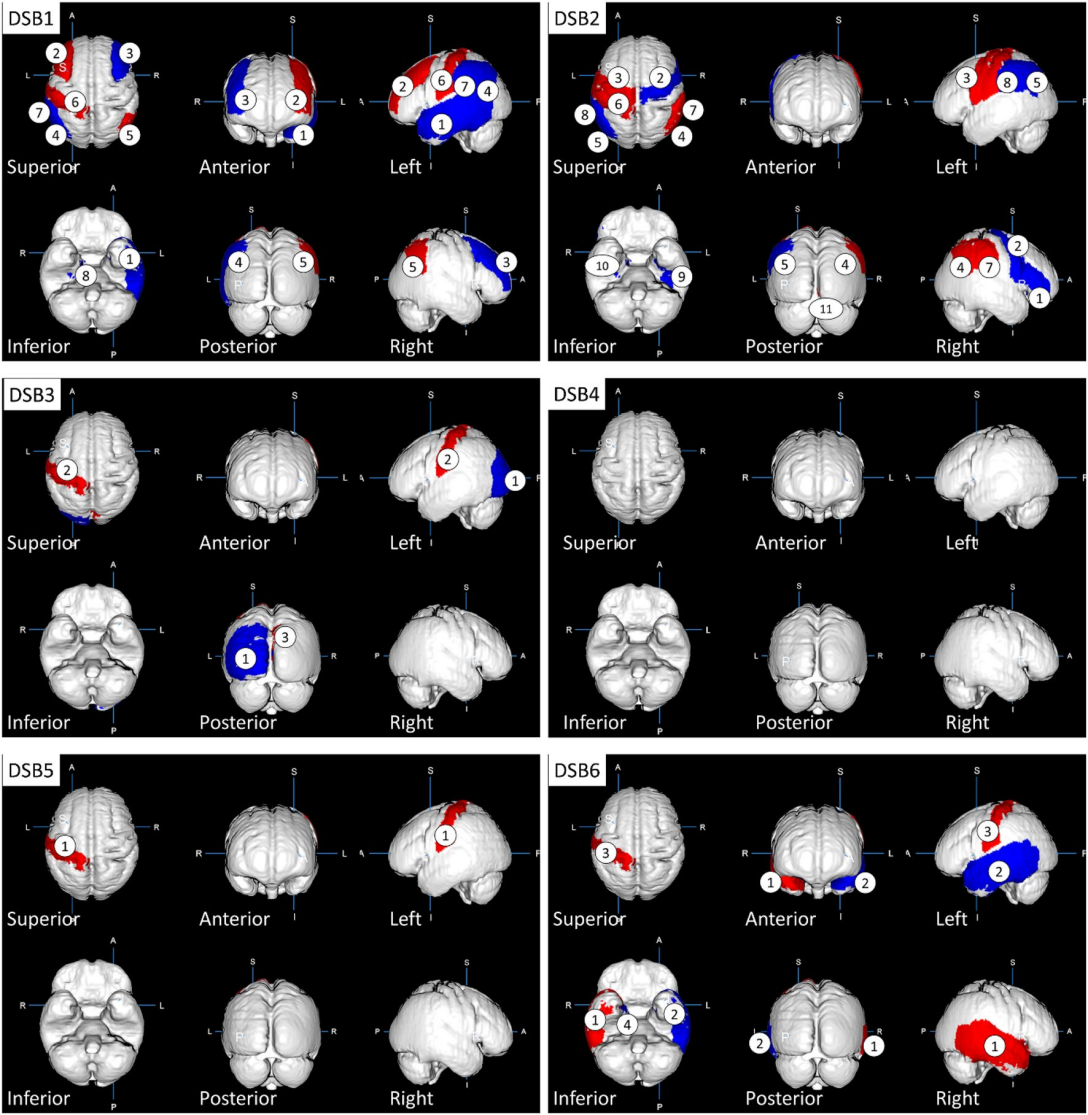
Regional gray matter regions positively and negatively involved in driving safety behaviors (DSBs). Mapping results with regards to the positive (blue) and negative (red) correlation of the regions with DSB1-6. The regional gray matters are as follows: [DSB1] ① left temporal gyrus, ② right middle frontal gyrus, ③ left middle frontal gyrus,④ right angular gyrus, ⑤ left angular gyrus, ⑥ left postcentral gyrus, ⑦left supramarginal gyrus, ⑧ right parahippocampal gyrus; [DSB2] ① right inferior frontal gyrus,② right precentral gyrus, ③ left precentral gyrus, ④ right angular gyrus, ⑤ left angular gyrus, ⑥ left postcentral gyrus, ⑦ right supramarginal gyrus, ⑧ left supramarginal gyrus, ⑨ left fusiform gyrus, ⑩ right parahippocampal gyrus, ⑪ right lingual gyrus; [DSB3] ① left occipital gyrus, ② left postcentral gyrus, ③ right cuneus; [DSB5] ① left postcentral gyrus; [DSB6] ① right temporal gyrus, ② left temporal gyrus, ③ left postcentral gyrus, ④ right entorhinal area.

The significantly correlated GM regions are shown in Fig. 2. The correlated parts are colored blue and red for positive and negative correlation, respectively. “Positive” means higher volumes related to higher scores in a respective DSB category, whereas “negative” means lower volumes related to higher scores in the respective DSB category.

Notably, the left postcentral gyrus displayed negative correlations with five DSB categories, while the left temporal, left angular, left supramarginal, and right parahippocampal gyrus were positively correlated with two DSB categories. The right angular gyrus was negatively correlated with two DSB categories. Additionally, the right inferior and middle frontal gyrus, precentral gyrus, entorhinal area, left occipital, and fusiform gyrus exhibited positive correlations with one DSB category. The right temporal, lingual, supramarginal gyrus, cuneus, and the left middle frontal and precentral gyrus displayed negative correlations with one DSB category. When a GM region correlated with multiple DSB categories, all correlations were positive or negative, which indicates a uniform determination of positive or negative for a GM region (Table 4).

Crucially, post Bonferroni and FDR corrections, only the left postcentral gyrus retained statistical significance with DSB-1 (visual search behavior) as can be seen in Table 3, emphasizing the necessity to interpret results with caution, particularly concerning the robustness of associations between GM regions and DSB categories.

## Discussion

### Uniform determination of positive or negative correlation

Initially, we speculated that the volumes of different GM regions are positively correlated with the scores of different DSB categories, because brain atrophy causes both mental and physical decline resulting in the degradation of safe driving skills, such as visual and spatial cognition, attention, decision-making, and execution ability^21–23^. Nevertheless, this study revealed a regularly positive or negative correlation of each GM region with DSBs without exception. Ten of 56 GM regions were positively correlated with DSBs, while 8 GM regions were negatively correlated (Table 4). The negative reaction, which is contrary to the initial speculation, may be due to inefficiency and dedifferentiation often reported in aging brains^24^. It may alternatively be involved in a biological defense reaction. We previously examined the relationship between fatigue perception intensity and regional GM volumes in healthy subjects and found that there are regions where GM volume increases for compensation as fatigue increases^25^. In any case, there is no doubt that the relationship between DSB and GM region is more complex than expected. Furthermore, this finding suggests the possible existence of a brain mechanism for DSBs in which two GM regions with positive or negative correlations are uniformly organized regardless of the categories. If the positive or negative volumetric reactions reflect the hyperactivity or hypoactivity of neuronal functions between GM regions, the neuronal network for DSBs may contain the interaction between positive and negative responses in the complex space of the brain, in other words, a complex of two opposite directions, i.e., dual mechanics such as Yin and Yang. In the next step, a functional MRI (fMRI) study is planned to elucidate the neuronal connectivity regulating DSBs^26^.

### Gender differences

Considering the overall results in Tables 1 and 2, some DSB categories responded negatively to brain volumes for the female participants compared with the male participants. A difference in the brain structure of males and females may have caused this effect^27^. Gender differences may have a greater impact on the whole brain, GM, WM, and cerebral lobes than on GM regions, because the significance disappeared in the regression analysis of the 56 GM regions. This may be the reason why GM regions are too small to detect gender differences for DSBs. Further verification using large samples is necessary because of the established gender difference in brain structures.

### Differences in DSB reactions

The number of significantly correlated GM regions varies by DSB categories: 11 GM regions for speeding (DSB2), 8 for visual search behavior (DSB1), 4 for steering (DSB6), 3 for signaling (DSB3), 1 for positioning (DSB5), and none for vehicle stability (DSB4). Thus, positioning and vehicle stability of DSBs, which are naturally essential for safe driving, were extremely low in correlation numbers. In this study, a participant drives a car with an official instructor sitting next to him/her, and the nervous situation may have largely affected DSB5 and DSB4 compared with other categories of DSBs. Most participants carefully drove approximately 20 km/h (data not shown) on the straight course, except for the six checkpoints. This slow speed or careful driving behaviors may especially correlate with none or only one of the GM regions for DSB4 or DSB5, respectively. The advantage of this study is the investigation using six categories of DSBs when driving an actual motor vehicle on a closed circuit. However, the experimental condition is largely different from free driving in residential areas. This difference may have degaussed the variation in the evaluation of DSBs. Further validation with free driving in residential areas is necessary.

### Relationship between the GM region and DSBs

The number of significantly correlated DSB categories varies by GM regions: 5 categories for the left postcentral gyrus, 2 for the right angular and parahippocampal gyrus and the left temporal, angular, and supramarginal gyrus, and 1 for 12 GM regions. Thus, the left postcentral gyrus may play an important role in the sufficient enforcement of DSBs because of the higher frequency although it was negatively correlated with DSBs and belongs to the somatosensory cortex unrelated to motor function^28^. Nevertheless, two previous studies have shown completely different results: the study by the Toyota Central Institute reported the supplemental motor area^15^ and another by Keio University revealed the four GM regions, namely, the left superior part of the precentral sulcus, left sulcus intermedius primus, right orbital part of the inferior frontal gyrus, and right superior frontal sulcus^16^. Thus far, no studies except for the two are targeting the relationship between regional GM volumes and driving performances. Therefore, research outcomes should be evaluated carefully because the correlated brain regions may largely change depending on the experimental conditions, kinds of motor vehicles, driving on a closed-circuit course or free driving, driving locations, and methods of DSB evaluation.

GM gyri multiply correlated to DSB categories are the following; the left postcentral, angular, right parahippocampal, left temporal, and left supramarginal ones. Regardless of girth or left, the postcentral gyrus is a primary somatosensory area and it may be involved in the fine adjustment of steering wheel and pedal operations. The angular gyrus plays an important role in attention, spatial cognition, and semantic information processing^29^. The parahippocampal gyrus is a cortex region in the medial temporal lobe that surrounds the hippocampus and plays an important role in both spatial memory^30^ and navigation^31^. The temporal gyrus serves as multimodal brain functions for language, auditory processing, navigation, and comprehension^32^. The supramarginal gyrus is part of the somatosensory association cortex and is involved in motor planning and execution^33^. These descriptions provide plausible explanations for the relationship between GM regions and DSB categories although left and right specificity may affect DSB.

### Right–left symmetry

The correlated GM regions with right–left symmetry were temporal, middle frontal, precentral, angular, and supramarginal gyrus (Table 4). Interestingly, right–left symmetry has a combination of positive and negative responses, that is, the middle frontal, precentral, and angular gyrus were positively or negatively correlated in the right or left cerebral hemisphere, respectively. On the other hand, the temporal and supramarginal gyrus were positively or negatively correlated in the left or right cerebral hemispheres, respectively. The results indicated that associated fibers between the right and left hemispheres may regulate the dual mechanics in brain functions for DSBs.

### Utilization of DSB and brain data

Noninvasive brain-imaging techniques, such as near-infrared spectroscopy and electroencephalography, can allow measurements of brain activations within actual motor vehicles but remain challenging in the reduction of motion and electric noises^34^. In this study, volumetric data obtained by MRI were used for analyses. Recently, an fMRI study showed that sensorimotor areas increase their activities after changing the direction of a virtual car on a monitor on the bed of an MRI scanner^35^. If fMRI data can be additionally utilized, it will be possible to reproducibly identify brain regions involved in DSBs from both structural and functional data of the brain although MRI cannot measure in real-time on roads. Older drivers who may drive dangerously could be identified with MRI in advance when renewing their driving license soon. Brain atrophy is largely dependent on lifestyles, smoking, drinking alcohol, less exercise, and lack of sleep^13^. The improvement of lifestyles may not only affect the whole brain but also the cerebral regions and lead to the upregulation of driving performances.

### Limitations

The interpretation of our study results should be approached with caution due to several inherent limitations. Firstly, the relatively small size of our participant cohort may affect the generalizability of findings. Despite being comparable to or even larger than some prior studies^15,16^, this limitation underscores the need for larger sample sizes in future investigations.

Secondly, the integration of actual driving evaluation and MRI, while offering valuable insights, presents inherent challenges in terms of feasibility. Balancing these two diverse methodologies requires careful consideration, and our study might benefit from advancements in experimental design that address potential confounding factors more effectively.

Thirdly, the controlled environment of the closed-circuit course under the supervision of an instructor, while beneficial for experimental control, may not fully capture the nuances of real-world driving conditions. This discrepancy may influence the assessment of certain DSB categories, particularly positioning and vehicle stability.

Moreover, the absence of a systematic exploration of factors such as sleep, physical condition, and mood during the day represents a limitation. These factors are known to influence DSBs, and their unaccounted variability could impact the study's internal validity.

A critical consideration arises from the significant findings of the relationship between GM regions and DSBs. Post Bonferroni and FDR corrections, only the left postcentral gyrus retained statistical significance with DSB-1 (visual search behavior). This underscores the importance of acknowledging the impact of multiple comparisons on the interpretation of results and raises questions about the robustness of associations between GM regions and DSB categories.

Furthermore, the present study diverged from previous methodologies employed in the field, encompassing variations in locations, point scales for evaluation, and DSB categories. This departure hampers direct comparisons with earlier findings, emphasizing the importance of contextualizing our results within the specific parameters of our study.

Lastly, our participant pool exclusively comprised individuals over the age of 70. While this demographic choice aligns with the focus on older drivers, it raises questions about the generalizability of our findings to a broader age spectrum. To establish the universality of the observed brain-DSB relationships, future studies should encompass diverse age groups.

These limitations collectively highlight the nuanced nature of the brain-DSB relationship, necessitating careful consideration of contextual factors and the iterative refinement of methodologies in future investigations.

### Supplementary Information

Supplementary Table 1.

## Data Availability

The data supporting the findings of this study are available upon request from the corresponding author.
