# Supplementary material for: Uniformly positive or negative correlation of cerebral gray matter regions with driving safety behaviors of healthy older drivers
Source: Sci Rep. 2024 Jan 2;14:206. doi: 10.1038/s41598-023-50895-7 (PMC10762062; doi:10.1038/s41598-023-50895-7)
Supplement: Supplementary file 1 — Supplementary Table 1. [file 41598_2023_50895_MOESM1_ESM.docx]

**Supplementary 1**. Collinearity test result, including variance inflation factor (VIF) parameter of the 18 significantly correlated gray matter (GM) regions.

| **Explanatory variable** | **Collinearity Statistics** | |
| --- | --- | --- |
|  | **Tolerance** | **VIF** |
| (Constant) |  |  |
| Sex (Male:0; Female:1) | 0.729 | 1.371 |
| Age | 0.726 | 1.378 |
| Right Inferior Frontal Gyrus | 0.414 | 2.417 |
| Right Temporal Gyrus | 0.159 | 6.270 |
| Left Temporal Gyrus | 0.210 | 4.756 |
| Left Occipital Gyrus | 0.322 | 3.107 |
| Right Middle Frontal Gyrus | 0.217 | 4.610 |
| Left Middle Frontal Gyrus | 0.251 | 3.988 |
| Right Precentral Gyrus | 0.188 | 5.330 |
| Left Precentral Gyrus | 0.167 | 6.000 |
| Right Angular Gyrus | 0.304 | 3.285 |
| Left Angular Gyrus | 0.283 | 3.532 |
| Left Postcentral Gyrus | 0.308 | 3.246 |
| Right Supramarginal Gyrus | 0.431 | 2.319 |
| Left Supramarginal Gyrus | 0.272 | 3.682 |
| Right Entorhinal area | 0.260 | 3.844 |
| Left Fusiform Gyrus | 0.440 | 2.275 |
| Right Parahippocampal Gyrus | 0.378 | 2.647 |
| Right Cuneus | 0.343 | 2.914 |
| Right Lingual Gyrus | 0.419 | 2.386 |
